# Impact of glycemia on survival of glioblastoma patients treated with radiation and temozolomide

**DOI:** 10.1007/s11060-015-1815-0

**Published:** 2015-05-27

**Authors:** Minh Thi Tieu, Leif E. Lovblom, Mairéad G. McNamara, Warren Mason, Normand Laperriere, Barbara-Ann Millar, Cynthia Ménard, Tim-Rasmus Kiehl, Bruce A. Perkins, Caroline Chung

**Affiliations:** Department of Radiation Oncology, University of Toronto/ University Health Network-Princess Margaret Cancer Centre, 610 University Ave, Toronto, ON M5T 2M9 Canada; Division of Endocrinology and Metabolism, University of Toronto/Leadership Sinai Centre for Diabetes, Mount Sinai Hospital, L5-210, 60 Murray Street, Mail Box 16, Toronto, ON M5T 3L9 Canada; University Health Network-Princess Margaret Cancer Centre, Neuro-oncology, 610 University Ave, Toronto, ON M5T 2M9 Canada; Institute of Cancer Sciences, University of Manchester/The Christie NHS Foundation Trust, Wilmslow Road, Withington, Manchester, M204BX UK; Department of Pathology, University Health Network, 200 Elizabeth St., Toronto, ON M5G 2C4 Canada

**Keywords:** Glioblastoma, Glucose, Glycemia, Radiation, Temozolomide, Prognostic

## Abstract

Evidence suggests hyperglycemia is associated with worse outcomes in glioblastoma (GB). This study aims to confirm the association between glycemia during radiotherapy (RT) and temozolomide (TMZ) treatment and overall survival (OS) in patients with newly diagnosed GB. This retrospective study included GB patients treated with RT and TMZ from 2004 to 2011, randomly divided into independent derivation and validation datasets. Time-weighted mean (TWM) glucose and dexamethasone dose were collected from start of RT to 4 weeks after RT. Univariate (UVA) and multivariable (MVA) analyses investigated the association of TWM glucose and other prognostic factors with overall survival (OS). In total, 393 patients with median follow-up of 14 months were analyzed. In the derivation set (n = 196) the median OS was 15 months and median TWM glucose was 6.3 mmol/L. For patients with a TWM glucose ≤6.3 and >6.3 mmol/L, median OS was 16 and 13 months, respectively (p = 0.03). On UVA, TWM glucose, TWM dexamethasone, age, extent of surgery, and performance status were associated with OS. On MVA, TWM glucose remained an independent predictor of OS (p = 0.03) along with TWM dexamethasone, age, and surgery. The validation set (n = 197), with similar baseline characteristics, confirmed that TWM glucose ≤6.3 mmol/L was independently associated with longer OS (p = 0.005). This study demonstrates and validates that glycemia is an independent predictor for survival in GB patients treated with RT and TMZ.

## Introduction

Glioblastoma (GB) is the most common malignant primary brain tumor in adults. Despite aggressive surgical resection and combined temozolomide chemotherapy and radiotherapy (RT), even in younger patients with good performance status, the median survival remains relatively poor at 14.6 months [1]. Therefore, novel approaches are required to improve outcomes for patients with this devastating diagnosis.

A number of factors have been identified to be prognostic for survival in GB patients including age, performance status, neurological function, neurocognitive function, extent of surgical resection, MGMT promoter methylation and IDH1 mutation [2–5]. These factors are typically considered when making recommendations regarding an individual’s initial treatment.

There is growing evidence that blood glucose and glucose metabolism play an important role in cancer development and growth, including GB [6–12]. Specifically for GB, it has been reported that altered mechanisms of energy metabolism impact tumor growth [13]. The Warburg effect has been suggested as the mechanism for greater tumor dependence on glucose for growth in GB cells in vitro [14]. Under this hypothesis a high glucose environment may promote tumor growth and progression. In addition, associated insulin resistance and high circulating insulin may further promote tumor growth via insulin like growth factor (IGF) signaling pathways [15]. Studies suggest that patients with solid cancers, including breast and colon cancer, have worse survival if they also have pre-existing diabetes [16].

For patients with GB, two retrospective studies have shown an association between higher blood glucose levels and worse survival [17, 18]. However, these studies included heterogeneous patient populations who received a variety of treatments including various doses and schedules of RT and inconsistent treatment with temozolomide following surgery. In addition, these studies did not account for a major confounding factor, dexamethasone use, and evaluated blood glucose levels at variable time points with various approaches.

This study aimed to investigate the association between glycemia and survival in GB patients treated with standard of care six-week course of RT concurrently with temozolomide following surgery, and was designed to quantitatively account for major confounding factors including dexamethasone dose and to incorporate both a derivation and validation set.

## Materials and methods

This was a single institution retrospective study with independent derivation and validation cohorts of patients treated at a tertiary cancer centre for histologically-confirmed GB. Patients were treated between January 2004 and June 2011 with definitive RT to a total dose of 54 to 60 Gy in 30 daily weekday fractions of 1.8 to 2 Gy per day using conformal techniques in combination with concurrent temozolomide 75 mg/m^2^ daily. This was generally followed with adjuvant temozolomide 150–200 mg/m^2^. Variability in clinical approaches to therapy was not observed over the course of the study period. Patients were excluded if they had no blood glucose documentation, received other radiation doses or fractionation schedules or received no concurrent temozolomide.

After receiving institutional research ethics approval, an electronic chart review was completed to collect the following baseline patient and tumor characteristics: age at treatment, body mass index (BMI), prior history of diabetes and use of diabetic medication, Eastern Co-operative Group (ECOG) performance status, and isocitrate dehydrogenase 1 (IDH-1) mutation status. The following treatment factors were collected: extent of surgery, radiation dose fractionation and temozolomide treatment details. At our institution, random blood glucose levels were measured routinely at the time of surgery, prior to RT, weekly during the course of RT and monthly during adjuvant temozolomide. Dexamethasone doses were also recorded at the time of initial consultation, regularly during RT and in follow-up after completion of RT. For each patient, time weighted mean (TWM) values were calculated from start of RT to 4 weeks following completion of RT (week 10) for serial blood glucose measurements (TWM glucose) and dexamethasone doses The calculation of TWM values incorporates multiple measures between a pre-determined time period (i.e. 10 weeks for this study) and accounts for the time between each measure. This method helps account for the effects of variability in these measures over the 10-week period. For dexamethasone, this approach helps determine the most representative average dexamethasone exposure over the 10 weeks, accounting for titration of dose during this period to manage symptoms. For glucose levels, this approach helps account for changes in glucose level over the 10-week period and helps reduce the impact of variability in glucose levels when they are taken randomly as opposed to consistent fasting glucose readings.

The formula to calculate time-weighted means is as follows:

$$\frac{{\mathop \sum \nolimits_{i}^{n} \omega_{i} x_{i} }}{{\mathop \sum \nolimits_{i}^{n} \omega_{i} }}$$

where ω = the proportion of days until either the next measurement or the 28th day following RT and *x* = either blood glucose measurements or dose of dexamethasone, whichever is being calculated.

### Statistical analysis

Study subjects were randomly divided into two subgroups, the Derivation Set and the Validation Set, using Simple Random Sampling in order to minimize any bias associated with changes in practices and reflected outcomes over time. Differences in baseline characteristics between the two sets were assessed using linear regression for continuous parametric variables, the Wilcoxon rank-sum test for nonparametric continuous variables, and logistic regression for dichotomous and categorical variables.

Survival time was calculated as the time between diagnosis of the tumor and the date of death; observations were censored if the subject was alive at the date of last contact. The effect of glycemia on overall survival in the Derivation Set was assessed using a Cox proportional hazards model. The Derivation Set was then divided into two groups based on the subjects’ TWM glucose, one group with glucose less than or equal to the median, the other with glucose greater than the median. Univariate Cox regression was also used to evaluate the effects of other variables on survival, and those with significant p-values were then included in a multivariable model to determine the independent association of glucose and overall survival. This process was then repeated in the Validation Set, using the same glucose cut-off as the Derivation Set. Kaplan–Meier survival curves were generated for both sets, with subjects divided by their TWM glucose. Statistical analysis was performed using SAS version 9.3 for Windows (SAS Institute, Cary, North Carolina), and p-values less than 0.05 were considered significant.

## Results

### Total cohort

A total of 393 patients met eligibility criteria and were included in the final analysis. The entire cohort had a mean age of 54 years. Median follow up for the study population was 14 months (1–104 months) and median survival was 15 months. The majority of patients (86 %) had a performance status of ECOG 0–1. The majority of patients (91 %) also received dexamethasone during treatment. Only 9 % of patients had a prior diagnosis of diabetes mellitus and mean BMI was 27.2 kg/m^2^. The mean number of glucose readings per patient was 5 ± 2. The IDH-1 mutation status was confirmed for 127 (32 %) cases of which 7 (6 %) were IDH-1 mutation positive.

Patients were divided randomly into a derivation set of 196 patients and a validation set of 197 patients. Baseline characteristics of the derivation and validation set are summarized in Table 1. The derivation and validation cohorts were similar with regards to patient age, BMI, performance status, extent of surgery and radiation dose fractionation regimens. Fewer patients in the validation set were on dexamethasone (88 vs. 95 %, p = 0.01). In the derivation and validation sets, 5/73 (7 %) patients and 2/54 (4 %) patients had IDH-1 positive tumors respectively.

**Table 1:** Patient baseline characteristics according to derivation and validation set

Characteristics	Set		P-value
	Derivation (n = 196)	Validation (n = 197)	
Age (average, years)	54	54	0.99
Female Gender, n (%)	71 (36 %)	71 (36 %)	0.97
BMI (kg/m^2^)	26.7	27.6	0.08
BMI ≥ 25, n (%)	126 (64 %)	122 (62 %)	0.94
Pre-existing diabetes	17 (9 %)	19 (10 %)	0.74
Mean glucose (mmol/L)	7.4	7.0	0.30
*ECOG performance status, n (%)*			
0–1	171 (87 %)	168 (85 %)	0.57
2–3	25 (13 %)	29 (15 %)	
Proportion of patients on Dexamethasone, n (%)	187 (95 %)	172 (88 %)	0.01
Mean TWM dexamethasone dose	4.5	4.0	0.03
*Extent of surgery, n (%)*			
Subtotal	35 (18 %)	37 (19 %)	0.76
Partial	120 (61 %)	122 (62 %)	
Biopsy	40 (20 %)	38 (19 %)	
Unknown	1 (1 %)	–	
*Radiation dose, n (%)*			
60/30	160 (82 %)	172 (87 %)	0.16
54/30	26 (13 %)	13 (7 %)	
Other	10 (5 %)	12 (6 %)	
Temozolomide n (%)	196 (100 %)	197 (100 %)	–
Concurrent	41 (21 %)	36 (18 %)	0.51
Concurrent & adjuvant	155 (79 %)	161 (82 %)	
Adjuvant cycle number	4	3	0.77
Metformin use	27 (14 %)	20 (10 %)	0.27
Hyperglycemia interventions	43 (22 %)	30 (15 %)	0.09
Salvage treatment, n (%)	114 (58 %)	119 (60 %)	0.65
Hospital admission, n (%)	86 (44 %)	82 (42 %)	0.65
Acute infection, n (%)	44 (22 %)	53 (27 %)	0.31

*BMI* body mass index, *ECOG* eastern cooperative oncology group, *TWM* time weighted mean

### Derivation set

**Fig. 1:**
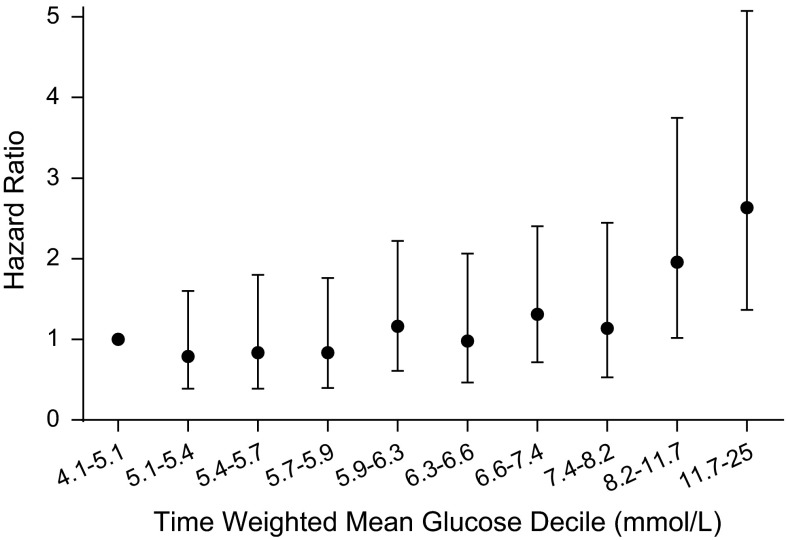
Hazard Ratio according to time weighted mean glucose decile as compared with first decile, in the derivation set. *Dot* shows hazard ratio and *error bars* represents 95 % confidence interval. Deciles are divided as follows: 4.1–<5.1 mmol/L, ≤5.1–<5.4 mmol/L, ≤5.4–<5.7 mmol/L, ≤5.9–<6.3 mmol/L, ≤6.3–<6.6 mmol/L, ≤6.6–<7.4 mmol/L, ≤7.4–<8.2 mmol/L, ≤8.2–<11.7 mmol/L, ≤11.7–25 mmol/L

**Fig. 2:**
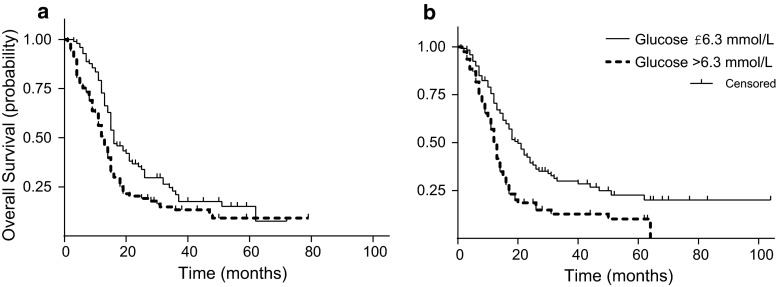
Kaplan Meier survival curves for the derivation set (**a**) and validation set (**b**) divided by time weighted glucose ≤6.3 and >6.3 mmol/L. Derivation set adjusted *p* value for trend = 0.03, validation set adjusted p-value for trend = 0.005

The derivation set included 196 patients with a median survival of 15 months. The relationship between TWM glucose and survival was initially examined in deciles. A univariate model revealed that the hazard ratio (HR) increased significantly above the median TWM glucose of 6.3 mmol/L, above the 5th decile (Fig. 1). Hence patients were divided into two groups, above and below this median TWM glucose. The median overall survival for patients with a TWM glucose of ≤6.3 and >6.3 mmol/L was 16 and 13 months, respectively (Fig. 2a). Patients with TWM glucose levels above 6.3 mmol/L were more likely to have pre-existing diabetes (14 vs. 3 %, p = 0.01), and less likely to have received adjuvant monthly temozolomide following concurrent radiation with temozolomide (67 vs. 91 %, p = 0.0001).

On univariate analysis (Table 2), factors that were associated with survival included TWM glucose, TWM dexamethasone dose, age, ECOG performance status and extent of surgery (biopsy vs partial/subtotal resection). Although patients with higher glucose levels were more likely to have pre-existing diabetes this factor was not associated with survival on UVA. On multivariable analysis, TWM glucose remained an independent predictor of survival such that a TWM glucose of >6.3 mmol/L had a hazard ratio for death of 1.47 (95 % CI: 1.05–2.06, p = 0.03) (Table 3). Other factors that were independently associated with survival on multivariable analysis included TWM dexamethasone dose (HR 1.04, 95 % CI: 1.05–2.06, p = 0.02), age (HR 1.02, 95 % CI: 1.01–1.04, p = 0.01) and type of surgery (biopsy vs. partial/subtotal, HR 0.64, 95 % CI 0.43–0.95, p = 0.03).

**Table 2:** Univariate association between patient characteristics and survival in derivation set

Characteristic	HR	95 % CI	P-value
*Median TWM glucose*			
≤6.3 mmol/L	Reference		
>6.3 mmol/L	1.59	(1.15,2.19)	0.005
Mean TWM dexamethasone dose, per mg	1.05	(1.02,1.08)	0.0005
Age, per year	1.02	(1.003,1.04)	0.02
BMI, kg/m^2^	1.00	(0.97,1.04)	0.76
Sex, female versus male	1.25	(0.90,1.75)	0.19
*ECOG*			
0 or 1	Reference		
2 or 3	1.88	(1.17,3.01)	0.009
Pre-existing Diabetes, no versus yes	1.50	(0.89,2.53)	0.13
Metformin, no versus yes	1.02	(0.65,1.61)	0.92
Surgery, biopsy versus partial/subtotal	0.57	(0.39,0.84)	0.004

*BMI* body mass index, *ECOG* eastern cooperative oncology group, *TWM* time weighted mean

**Table 3:** Multivariable association between patient characteristics and survival in derivation and validation sets

Characteristic	HR	95 % CI	P-value
*Derivation set*			
Median TWM glucose			
≤6.3 mmol/L	Reference		
>6.3 mmol/L	1.47	(1.05,2.06)	0.03
Mean TWM dexamethasone dose, per mg	1.04	(1.01,1.07)	0.02
*ECOG performance status*			
0 or 1	Reference		
2 or 3	1.45	(0.83,2.54)	0.19
Age, per year	1.02	(1.01,1.04)	0.01
BMI, kg/m^2^	0.99	(0.95,1.03)	0.57
Surgery, biopsy versus partial/subtotal	0.64	(0.43,0.95)	0.03
*Validation set*			
Median TWM glucose			
≤6.3 mmol/L	Reference		
>6.3 mmol/L	1.67	(1.17,2.40)	0.005
Mean TWM dexamethasone dose, per mg	1.08	(1.04,1.11)	<0.0001
*ECOG performance status*			
0 or 1	Reference		
2 or 3	1.55	(0.91,2.65)	0.11
Age, per year	1.03	(1.01,1.05)	0.01
BMI, kg/m^2^	1.04	(1.01,1.08)	0.02
Surgery, biopsy versus partial/subtotal	1.04	(0.64,1.67)	0.88

*BMI* body mass index, *ECOG* eastern cooperative oncology group, *TWM* time weighted mean

When higher glucose cut-off values were explored, survival was consistently worse for patients with glucose values above the cut-off, and the adjusted hazard ratio grew larger as higher cut-off values were used. In the derivation set, the cut-off of 6.3 mmol/L yielded an adjusted HR of 1.47; 6.6 mmol/L yielded an adjusted HR of 1.53; 7.4 mmol/L yielded an adjusted HR of 1.58; and 8.2 mmol/L yielded an adjusted HR of 1.88.

### Validation set

The validation set included 197 patients. Median survival for these patients was 16 months. The median TWM glucose for the validation set was 6.0 mmol/L. Patients were divided into two groups based on the glucose threshold from the Derivation set. Median survival for patients with a TWM glucose of ≤6.3 and >6.3 mmol/L was 20 and 13 months, respectively (Fig. 2b). Again, univariate analysis showed the following variables were associated with survival: TWM glucose, time weighted mean dexamethasone dose, age, BMI, ECOG performance status and extent of surgery (biopsy vs partial/subtotal resection). In this validation cohort, time weighted mean glucose of greater than or less than the previously defined median value of 6.3 mmol/L remained an independent prognostic factor for survival on multivariable analysis. The hazard ratio for death was 1.67 (95 % CI: 1.17–2.4, p = 0.005) for patients with a time weighted mean glucose of >6.3 mmol/L when compared with patients with ≤6.3 mmol/L (Table 3).

### Dexamethasone and glucose

The linear relationship between TWM glucose and TWM dexamethasone was found to be weak, with a Spearman rank-correlation coefficient of 0.20 (p < 0.0001).

## Discussion

Our study demonstrates an independent association between higher blood glucose levels and survival in newly diagnosed GB patients treated with concurrent RT and temozolomide. Through independent derivation and validation cohorts, with particular attention to confounding variables including quantitative exposure to dexamethasone, we demonstrated that patients with a TWM blood glucose >6.3 mmol/L have significantly worse survival compared with those patients whose TWM blood glucose was ≤6.3 mmol/L, with hazard ratios of 1.47 in the derivation cohort and 1.67 in the validation cohort. Other factors independently associated with survival on multivariable analysis included TWM dexamethasone dose (HR 1.04, 95 % CI: 1.05–2.06, p = 0.02), age (HR 1.02, 95 % CI: 1.01–1.04, p = 0.01) and type of surgery (biopsy vs. partial/subtotal, HR 0.64, 95 % CI 0.43–0.95, p = 0.03).

Higher dexamethasone requirements in patients may reflect patients with larger post-operative tumour burden and peritumoral edema, who are likely to have worse prognosis. But higher dexamethasone dose intake is also associated with increased insulin-resistance and this can result in higher glucose and insulin levels. While the analysis in this patient cohort suggests a weak association between dexamethasone and glucose in this clinical setting, it remains difficult to completely separate the impact of higher dexamethasone dose on glycemia and outcomes, given the known underlying physiological effects of corticosteroids on host glucose metabolism.

In our study, clinically meaningful differences in survival were observed with glucose levels above a glycemic threshold of 6.3 mmol/L, which is within the clinically accepted euglycemic range for random glucose. This low glycemic threshold has been confirmed internally in our Validation Set and is also consistent with the results of a study published in the pre-temozolomide era by Derr et al. [18]. This study divided glioblastoma patients into quartiles on the basis of TWM glucose taken from RT until last follow up date. It was reported that survival was poorer in patients in the higher quartile groups. The median TWM glucose in this study was 6.1 mmol/L. For patients with a TWM glucose of 6.1–7.6 mmol/L and >7.6 mmol/L median survival was 11.6 and 9.1 months, respectively. This was shorter than for patients with a TWM glucose of <5.2 mmol/L and 5.2–6.1 mmol/L where median survival was 14.5 and 11.6 months, respectively. In our study, the difference in median survival for patients above and below our glycemic threshold of 6.3 mmol/L was 3 months in the derivation set and 7 months in the validation set. These results are consistent with published data where the difference in survival between those with lower versus higher blood glucose levels ranged from 4.9 to 6 months [17, 18]. This difference in survival is clinically meaningful for patients with GB as their median survival is relatively short at approximately 14.6 months if they have good performance status and are treated with high dose RT and temozolomide [1].

In current practice, glycemic interventions have generally not been prioritized in patients undergoing treatment for GB. Glycemic interventions are often deferred until patients’ glucose levels are well beyond the renal threshold—i.e. blood glucose levels >12 mmol/L. The consistent finding of improved survival associated with lower blood glucose levels and large survival differences in our study and previously published literature, provides compelling evidence to motivate investigation of intensive glycemic intervention with a lower target glucose range to improve survival in patients with GB.

Several mechanisms may contribute to the association between glycemia and GB survival including role of glucose as a substrate and associated insulin levels. Preclinical studies have demonstrated the Warburg effect, preferential anaerobic metabolism through glycolysis even in the presence of sufficient oxygen, in GB cells and xenograft models [19–21]. As glycolysis requires more glucose to produce the same amount of energy, it is postulated that the preferential glycolytic metabolism of GB tumors results in greater glucose dependence. Dependence of GB cell lines on glucose has been demonstrated in preclinical experiments in which withdrawal of glucose resulted in extensive apoptosis in GB cell lines but not in normal human astrocytes [14]. Epidemiologic studies have reported that breast cancer, colorectal cancer and high grade glioma patients with type 2 diabetes have worse outcomes compared to their non-diabetic counterparts [12, 16]. In addition to hyperglycemia, patients with type 2 diabetes have hyperinsulinemia as a result of insulin-resistance. Higher circulating insulin levels may facilitate tumour growth through stimulation of the insulin-like growth factor (IGF) signalling pathways as insulin ligands have demonstrated high affinity to IGF-1 receptors [15, 22–24]. Glioblastoma cell lines have been shown to express IGF-1 receptors and laboratory studies have demonstrated that stimulation of the IGF-1 receptor results in GB tumor proliferation and migration [25].

Therefore, interventions that target lowering glucose levels as well as lowering insulin levels may improve outcomes of patients with GB. Recent studies have evaluated the potential for ketogenic diet approaches to reduce glucose levels. Strict administration of a ketogenic diet has been able to achieve blood glucose levels as low as 50–65 mg/dl (2.8–3.6 mmol/L), a range where induction of ketogenesis results. Laboratory experiments in animal models have suggested that a ketogenic diet and 2-deoxy-d-glucose administration impairs astrocytoma growth [26] and that this fasting state may sensitize glioma cells to RT and temozolomide [27]. To date, clinical data of ketogenic diets in GB patients are limited to a small retrospective review and case studies [28, 29]. Similarly, there have been small feasibility studies reported in metastatic cancer patients [30, 31]. Reports of tolerability of ketogenic diet in cancer patients are variable and larger trials for further assessment are warranted. In the GB patient population, alternative approaches for reducing blood glucose levels, such as oral diabetic agents may be more feasible than dietary restriction. We have planned a pilot study to assess the feasibility of early glycemic intervention with lower target glucose levels (4–7 mmol/L) in GB patients using diabetic agents including metformin, an agent that would potentially reduce both glucose and insulin levels.

Due to the retrospective nature of this study with inclusion of patients treated prior to the era of standard IDH-1 mutation testing; only 32 % of tumors had confirmed IDH-1 mutation results. Of the tumors that were tested only 6 % were found to harbor the IDH-1 mutation. The mechanism of through which IDH-1 mutation effect tumor outcomes is not well understood. One of the proposed mechanisms of relevance to our study is that GBM cells harboring the IDH mutation have impaired glucose oxidation leading to reduced production of energy and other substrates used for biosynthesis and resulting in slower growth rates [32, 33]. Based on this proposed mechanism, higher glucose exposure may not drive metabolism in tumors with IDH-1 mutations. However considering the small proportion of IDH-1 mutated tumors in our cohort, only 6 % of all evaluated tumors, the IDH mutations status of cases included in this study are unlikely to confound our findings.

As this is a retrospective study that included patients treated over a long time period, limitations of the study include the lack of O^6^-methylguanine–DNA methyltransferase (MGMT) methylation status and IDH-1 mutation status for only a portion of tumours. As the majority of our patients were treated prior to the era of recognizing pseudoprogression, we were unable to reliably assess the impact of glucose on local tumour progression, as patients received further treatment for radiological ‘progression’ shortly after completing concurrent temozolomide and RT. For these cases, we could not determine whether subsequent radiological improvement represented response to further treatment or resolution of pseudoprogression over time. A retrospective study has shown that patients with low-grade glioma who had persistent outpatient hyperglycemia (serum glucose >10 mmol/L on ≥3 occasions) had higher rates of tumor recurrence and lower survival [34]. Investigation of a more recent cohort of patients or prospective investigation of patients treated with concurrent radiation and temozolomide would help confirm the association between glycemia and tumor progression in patients with GB.

## Conclusion

Our findings demonstrate that glycemia is an independent predictor for survival in GB patients treated with RT and TMZ. This is independent of other known prognostic factors including age, extent of surgery, and dexamethasone requirements. In this study, incrementally lower glucose levels, even within the normal glycemic range, were associated with better survival. This motivates prospective clinical studies to investigate the effect of intense glycemic intervention during concurrent and adjuvant radiation and temozolomide therapy to maintain lower glucose levels in patients with glioblastoma.
